# Links between data on chemical and biological quality parameters in wastewater-impacted river sediment and water samples

**DOI:** 10.1016/j.dib.2018.05.068

**Published:** 2018-05-19

**Authors:** Miren Martínez-Santos, Anders Lanzén, Jessica Unda-Calvo, Iker Martín, Carlos Garbisu, Estilita Ruiz-Romera

**Affiliations:** a Department of Chemical and Environmental Engineering, University of the Basque Country, Plaza Ingeniero Torres Quevedo 1, E-48013 Bilbao, Basque Country, Spain; b Department of Conservation of Natural Resources, NEIKER-Tecnalia, Basque Institute of Agricultural Research and Development, Bizkaia Science and Technology Park, P 812, Berreaga 1, E-48160 Derio, Spain; c AZTI, Marine Research Division, Herrera Kaia, Portualdea z/g, E-20110 Pasaia, Basque Country, Spain; d IKERBASQUE, Basque Foundation for Science, Bilbao, Spain

**Keywords:** Wastewater, River sediment, Nutrients, Metals, Denitrifying genes, *Rhodocyclaceae*

## Abstract

In many urban catchments, the discharge of effluents from wastewater treatment plants (WWTPs), as well as untreated wastewaters (UWWs), presents a major challenge for the maintenance of river sediment and water quality. The discharge of these effluents cannot only increase the concentration of metals, nutrients and organic compounds in fluvial ecosystems, but also alter the abundance, structure and function of river bacterial communities. Here, we present data on chemical and biological quality parameters in wastewater-impacted and non-impacted river surface sediment and water samples. Overall, the concentration of nutrients (inorganic nitrogen) and some heavy metals (Zn, Ni and Cr) was positively correlated with the *nirS*/16S rRNA ratio, while *nirK*- and *nosZ*-denitrifier populations were negatively affected by the presence of ammonium in sediments. Bacterial community structure was significantly correlated with the (i) combined influence of nutrient and metal concentrations, (ii) the contamination level (non-impacted *vs*. impacted sites), (iii) type of contamination (WWTP or UWW), and (iv) location of the sampling sites. Moreover, the higher abundance of five genera of the family *Rhodocyclaceae* detected in wastewater-impacted sites is also likely to be an effect of effluent discharge. The data presented here complement a broader study (Martínez-Santos et al., 2018) [1] and they are particularly useful for those interested in understanding the impact of wastewater effluents on the abundance, structure and function of river bacterial communities involved in nitrogen cycling.

**Specifications Table**

**Table:** 

Subject area	*Environmental Science*
More specific subject area	*River ecosystems*
Type of data	*Chemical and biological data (Tables)*
How data was acquired	*Data were collected from river sediment and water samples*
Data format	*Analysed*
Experimental factors	*Sampling sites were chosen in an attempt to study the influence of wastewater effluents on river sediment and water quality, from the headwater (non-impacted sites) to the outlet of the catchment*
Experimental features	*Analysis of metabarcoding (16S rRNA gene) and qPCR (16S rRNA, nirK, nirS and nosZ genes) data; analysis of metals, nutrients and carbon in river sediments and water*
Data source location	*Deba River catchment (42.98182, − 2.56654), Basque Country, Spain*
Data accessibility	*Chemical and biological data are available in this article. Sequence data are available on*https://www.ncbi.nlm.nih.gov/bioproject/?term=PRJEB24857

**Value of the data**

• Data reveal that the abundance of *nirS*-denitrifiers, with respect to total bacteria (i.e. the *nirS*/16S rRNA ratio) was positively correlated with nitrogen concentration, while *nirK*- and *nosZ*-denitrifier populations were negatively affected by the presence of ammonium in sediments.
• Data show that wastewater effluents contained not only high amounts of nutrients (inorganic nitrogen and phosphate) but also heavy metals (Zn, Ni and Cu).
• Data reveal the influence of contamination level, chemical parameters and location of sampling sites on bacterial community structure.
• Data show the combined influence of nutrients and metal concentrations affect bacterial community structure.
• The higher abundance of five genera of the family *Rhodocyclaceae* detected on wastewater impacted sites highlights the effect of discharged effluents on sediment bacterial community structure.

## 1 Data

Our aim was to study the impact of anthropogenic contamination from wastewater effluents on the abundance, structure and function of bacterial communities present in surface sediments on the Deba River catchment. To this aim, chemical and biological quality parameters data were measured in river surface sediments and water samples [1].

### 1.1 Correlations between chemical and biological parameters

Table 1 shows the Spearman׳s correlations obtained between chemical and biological quality parameters from river water and surface sediment samples. Inorganic nitrogen (N-NO_3_^−^, N-NO_2_^−^ and N-NH_4_^+^) and phosphorous (P-PO_4_^3^^−^) were significantly correlated in river water. Similarly, nutrients were positively correlated with organic and inorganic carbon (DOC and TIC) and heavy metals (Zn, Ni and Cu) in surface sediments. 16S rRNA, *nirS* and *nosZ* gene copy abundances were significantly correlated among them. The *nirS*/16S rRNA ratio correlated positively with N-NH_4_^+^ and N-NO_2_^−^ concentrations in river water, as well as with sediment Zn, Ni and Cr concentrations. Instead, the abundance of *nirK*- and *nosZ*-denitrifiers, with respect to total bacteria (i.e. *nirK*/16S rRNA and *nosZ*/16S rRNA ratios) was negatively affected by sediment N-NH_4_^+^ concentration.

**Table 1:** Spearman׳s correlation coefficients (r) among chemical (N = 8) and biological (N = 9) parameters of river surface sediments and water from all sampling sites. Correlation is significant (p) at the 0.01 level for bold numbers and at 0.05 for italic and bold numbers.

			**1**	**2**	**3**	**4**	**5**	**6**	**7**	**8**	**9**	**10**	**11**
Surface sediment	16 S rRNA	**1**	1.00										
	*nirK*	**2**	0.47	1.00									
	*nirS*	**3**	***0.78***	0.55	1.00								
	*nosZ*	**4**	***0.70***	***0.78***	***0.72***	1.00							
	*nirK/16 S*	**5**	-0.38	0.53	-0.15	0.18	1.00						
	*nirS/16 S*	**6**	-0.17	0.08	0.42	0.02	0.30	1.00					
	*nosZ/16 S*	**7**	-0.35	0.38	0.08	0.25	***0.79***	0.52	1.00				
	*nirK/nirS*	**8**	-0.38	0.27	-0.52	0.03	***0.70***	-0.25	0.23	1.00			
	*nosZ/nirS*	**9**	-0.55	0.07	-0.63	-0.07	0.63	-0.32	0.50	***0.77***	1.00		
	TOC	**10**	0.52	0.40	-0.10	0.38	-0.06	***-0.83***	-0.45	0.33	0.17	1.00	
	TIC	**11**	0.57	-0.14	***0.74***	0.17	**-0.84**	0.38	-0.43	**-0.95**	**-0.91**	-0.26	1.00
	TOC/TIC	**12**	-0.19	0.38	-0.57	0.00	0.56	-0.62	0.00	**0.88**	0.62	0.64	***-0.81***
	TON	**13**	0.55	0.17	-0.02	0.07	-0.40	***-0.71***	-0.62	-0.05	-0.12	***0.76***	0.17
	NO_3_-N	**14**	0.31	-0.38	0.38	0.19	-0.57	0.07	-0.10	-0.57	-0.19	-0.29	0.52
	NH_4_-N	**15**	0.62	-0.14	0.36	0.17	**-0.91**	-0.24	***-0.76***	-0.52	-0.52	0.21	***0.71***
	Fe	**16**	0.19	-0.24	0.31	-0.26	-0.46	0.31	-0.48	-0.52	***-0.83***	-0.14	0.60
	Mn	**17**	0.19	-0.17	0.45	0.26	-0.08	0.38	0.31	-0.52	-0.24	-0.29	0.29
	Zn	**18**	-0.07	-0.33	0.38	-0.19	-0.34	***0.83***	-0.10	-0.62	***-0.76***	-0.62	0.62
	Ni	**19**	-0.19	-0.48	0.29	-0.29	-0.30	**0.86**	-0.02	-0.60	-0.67	***-0.71***	0.57
	Cu	**20**	0.52	0.12	0.69	0.12	-0.63	0.38	-0.31	**-0.86**	**-0.86**	-0.19	**0.91**
	Pb	**21**	0.14	0.05	-0.05	0.05	-0.14	-0.21	-0.55	0.24	-0.26	0.38	-0.07
	Cr	**22**	-0.40	-0.02	0.17	-0.31	0.28	**0.88**	0.43	-0.29	-0.38	-0.69	0.19
Water	DOC	**23**	-0.17	-0.07	-0.12	-0.31	0.31	-0.10	0.29	0.02	0.12	-0.07	-0.17
	PO_4_^3^-P	**24**	0.45	-0.29	0.67	0.10	-0.67	0.43	-0.21	**-0.95**	***-0.79***	-0.33	**0.88**
	NO_3_-N	**25**	0.31	-0.33	0.52	-0.05	-0.53	0.45	-0.31	***-0.76***	**-0.86**	-0.26	***0.71***
	NO_2_-N	**26**	0.10	-0.19	0.62	0.05	-0.40	**0.86**	0.14	***-0.83***	-0.67	***-0.74***	***0.76***
	NH_4_-N	**27**	0.02	-0.31	0.55	-0.02	-0.43	***0.81***	0.12	***-0.79***	-0.60	***-0.81***	***0.74***

			**12**	**13**	**14**	**15**	**16**	**17**	**18**	**19**	**20**	**21**	**22**	**23**	**24**	**25**	**26**	**27**
Surface sediment	16 S rRNA	**1**																
	*nirK*	**2**																
	*nirS*	**3**																
	*nosZ*	**4**																
	*nirK/16 S*	**5**																
	*nirS/16 S*	**6**																
	*nosZ/16 S*	**7**																
	*nirK/nirS*	**8**																
	*nosZ/nirS*	**9**																
	TOC	**10**																
	TIC	**11**																
	TOC/TIC	**12**	1.00															
	TON	**13**	0.24	1.00														
	NO_3_-N	**14**	***-0.76***	-0.02	1.00													
	NH_4_-N	**15**	-0.38	0.60	0.48	1.00												
	Fe	**16**	-0.31	0.10	-0.17	0.21	1.00											
	Mn	**17**	-0.62	-0.33	0.60	-0.19	0.05	1.00										
	Zn	**18**	-0.62	-0.45	0.05	0.10	0.69	0.26	1.00									
	Ni	**19**	-0.69	-0.52	0.17	0.05	0.60	0.36	**0.98**	1.00								
	Cu	**20**	-0.62	0.29	0.21	0.62	0.62	0.05	0.55	0.45	1.00							
	Pb	**21**	0.48	0.07	-0.57	0.02	0.50	-0.40	0.19	0.05	-0.07	1.00						
	Cr	**22**	-0.31	-0.60	-0.33	-0.43	0.50	0.14	***0.76***	***0.74***	0.33	0.02	1.00					
Water	DOC	**23**	0.00	0.23	-0.02	-0.45	0.21	0.33	-0.21	-0.17	-0.10	-0.21	0.11	0.18				
	PO_4_^3^-P	**24**	**-0.91**	0.05	0.67	0.45	0.52	0.67	0.57	0.60	***0.71***	-0.29	0.21	***0.83***	1.00			
	NO_3_-N	**25**	-0.64	-0.07	0.26	0.19	*0.83*	0.57	***0.74***	***0.71***	0.57	0.19	0.43	0.49	***0.83***	1.00		
	NO_2_-N	**26**	**-0.91**	-0.43	0.48	0.24	0.36	0.48	***0.81***	***0.83***	0.67	-0.33	0.60	0.65	***0.76***	0.60	1.00	
	NH_4_-N	**27**	**-0.93**	-0.48	0.57	0.26	0.31	0.45	***0.76***	***0.81***	0.60	-0.36	0.52	0.67	***0.74***	0.55	**0.98**	1.00

### 1.2 Biological data analysis

Data on the influence of chemical parameters, location of sampling site and contamination level of river surface sediment on the structure of sediment bacterial communities are shown in Table 2. Permutational Multivariate Analysis of Variance (PERMANOVA) reveals that, among all the chemical parameters studied here, Cu concentration and the TOC/TIC ratio had the highest influence on bacterial community structure. On the other hand, the location of the sampling site and the presence of wastewater effluents, independently of the type of effluent (WWTP or UWW, see Table 2) significantly affected the spatial distribution of bacterial communities in the Deba river catchment. To a lesser extent, the residual contamination level (WWTP or UWW) also affected bacterial community composition. Table 3 shows the results from permutation-based Mantel tests and partial Mantel tests performed to evaluate the influence of heavy metals and nutrients (inorganic nitrogen and phosphorous) on bacterial community structure. Metal concentrations influenced community structure independently of nutrient concentrations but, at the same time, the combined influence of both correlated significantly with community structure. Although nutrient concentrations did not result in significant PERMANOVA models (Table 2), Mantel tests indicate that the simultaneous presence of metals and nutrients had an effect on the structure of bacterial communities. Table 4 shows the abundance of genera of the family *Rhodocyclaceae* in sampling sites immediately upstream or downstream of wastewater discharge points. Five genera of this family, which contains many denitrifiers, were significantly more abundant downstream of wastewater discharge points: *Thaurea, Candidatus Accumulibacter, Denitratisoma, Propionivibrio* and *Ferribacterium*. The higher abundance of these genera in wastewater-impacted sites might reflect the effect of effluents on the structure of surface sediment bacterial communities present in the Deba River catchment.

**Table 2:** Permutational multivariate analysis of variance using distance matrices (PERMANOVA). Only models where all variables were evaluated as significant (according to *F*-tests) are listed. Asterisks indicate the least significant explanatory variable (**<0.01, *< 0.05). Sampling sites impacted by wastewater treatment plant (WWTP) and untreated wastewater (UWW) effluents. Chemical parameters: total inorganic and organic carbon (TIC and TOC) and Cu concentration. Chemical parameters were not measured in D7 sampling site, only biological ones [1].

**Samples**	**Explanatory variable**	**Residual/total degrees of freedom**	***R***^**2**^**(16S rRNA)**
All	Location	6/8	0.47**
All	Residual contamination level (from WWTPs or UWWs)	6/8	0.41*
All	Non-impacted *vs*. impacted (WWTPs or UWWs)	7/8	0.33**
All except D7	TIC	6/7	0.35*
All except D7	TOC/TIC	6/7	0.42**
All except D7	(1) Total inorganic carbon	5/7	0.55*
	(2) Non-impacted *vs*. impacted		
All except D7	Cu	6/7	0.41**

**Table 3:** Results from Mantel tests and partial Mantel tests evaluating the influence of heavy metals and nutrients on bacterial community structure.

**Explanatory variables**	**Conditioning variables**	**Dependent variables**	***R* statistic**	**Significance**
Metal concentrations	(None)	Prokaryotic community composition	0.60	*p* = 0.002
Nutrient concentrations	(None)	Prokaryotic community composition	0.60	*p* = 0.01
Metal concentrations	Nutrient concentrations	Prokaryotic community composition	0.45	*p* = 0.02

**Table 4:** Average abundance of genera of the family *Rhodocyclaceae* across all samples, and immediately upstream or downstream of WWTP or UWW. Only genera with average abundance > 0.01% are included.

**Genus**	**All samples**	**Upstream**	**Downstream**
*Thauera*	0.05%	0.04%	0.06%
*Ca. Accumulibacter*	0.27%	0.22%	0.60%
*Uliginosibacterium*	0.16%	0.19%	0.13%
*Denitratisoma*	0.08%	0.06%	0.22%
*Propionivibrio*	0.17%	0.24%	0.36%
*Ferribacterium*	0.07%	0.04%	0.24%

## 2 Experimental design, materials and methods

Samples of river surface sediments and water were taken from eight different sites along the Deba River catchment: from the headwater to the outlet of the catchment (more details of sampling sites in [1]). Sediment sub-samples (0–5 cm depth) were randomly collected using a sterilized plastic spoon. Samples were sieved (< 2 mm) in the field, according to USEPA method [2], and stored in two sterile polypropylene containers for chemical and biological analysis, respectively. Water samples were also collected in polyethylene bottles at all sampling points.

*Analysis of chemical parameters*. Surface sediments were air-dried and homogenized. Then, their moisture content was determined. Chemical analyses were performed in the dried sediment: total organic (TOC) and inorganic (TIC) carbon, inorganic nitrogen (N-NH_4_^+^ and N-NO_3_^−^) according to [3], and pseudo-total metal concentrations (Cu, Cr, Ni, Pb, Zn, Mn and Fe) after acid digestion. Water samples were filtered (0.45 μm) and chemical parameters were analysed [4]: dissolved organic carbon (DOC) and nutrients (N-NH_4_^+^, N-NO_3_^−^, N-NO_2_^−^ and P-PO_4_^3^^−^).

*Analysis of biological parameters*. Sediment samples for DNA analysis were stored fresh at − 20 °C. DNA was extracted from sediment samples (0.25 g of dry weight sediment) using Power Soil™ DNA Isolation Kit. Metabarcoding (amplicon) library preparations were carried out as described in [5]. The 16S rRNA gene was amplified from prokaryotes (primers information are available on [1]). Real-time qPCR (qPCR) was carried out for measurements of total bacteria (16S rRNA gene), *nirK, nirS* and *nosZ* gene copy abundance as described in [6]. qPCR conditions and primers are described in [1].

*Statistical analysis*. All analyses were performed using R/vegan [7] and SPSS software for Windows 20.0 (SPSS, Inc).
