# Supplementary material for: Links between data on chemical and biological quality parameters in wastewater-impacted river sediment and water samples
Source: Data Brief. 2018 May 19;19:616–22. doi: 10.1016/j.dib.2018.05.068 (PMC5997898; doi:10.1016/j.dib.2018.05.068)
Supplement: Supplementary file 1 — Transparency document [file mmc1.docx]

On the behalf of all authors, the corresponding author (Miren Martínez-Santos) confirms that there are no conflict of interests; therefore, the statement 'Declarations of interest: none' has been included in the end of manuscript.

Miren Martínez-Santos
